# Prevalence of Autonomic Dysreflexia in Patients with Spinal Cord Injury above T6

**DOI:** 10.1155/2017/2027594

**Published:** 2017-10-26

**Authors:** Eun Sun Lee, Min Cheol Joo

**Affiliations:** ^1^Department of Rehabilitation Medicine, Wonkwang University School of Medicine, 895 Muwang-ro, Iksan, Jeonbuk 54538, Republic of Korea; ^2^Department of Rehabilitation Medicine and Institute of Wonkwang Medical Science, Wonkwang University School of Medicine, 895 Muwang-ro, Iksan, Jeonbuk 54538, Republic of Korea

## Abstract

**Objective:**

To investigate the prevalence of autonomic dysreflexia (AD) using ambulatory blood pressure monitoring (ABPM) and the autonomic dysfunction following spinal cord injury (ADFSCI) questionnaire in patients with spinal cord injury (SCI) above T6.

**Methods:**

Twenty-eight patients diagnosed with SCI above T6 were enrolled. ABPM and ADFSCI were utilized to assess AD. Using ABPM, systolic blood pressure (SBP), diastolic blood pressure, and heart rate (HR) were measured at 30-minute intervals. AD was defined as SBP 20 mmHg higher than basal SBP, and the number of AD events was counted. The ADFSCI questionnaire evaluates the severity and frequency of the AD symptoms.

**Results:**

According to the ABPM, AD occurred in 26 patients and AD events occurred 5.8 ± 4.7 times. Average daytime and nighttime SBP were 119.9 ± 18.8 mmHg and 123.8 ± 21.2 mmHg, respectively, and the nighttime mean SBP appeared to be 4 mmHg higher than daytime mean SBP. These findings suggest the loss of nocturnal BP dipping in SCI patients. ADFSCI results revealed that 16 of the patients evaluated were symptomatic while 12 were asymptomatic.

**Conclusion:**

AD following SCI above T6 was highly prevalent and several patients seemed asymptomatic. These results suggest the necessity of proper diagnostic and therapeutic interventions for managing AD.

## 1. Introduction

Autonomic dysreflexia (AD) is a disease that commonly occurs in patients with spinal cord injury (SCI), especially in the cervical and upper thoracic level above T6 [1, 2]. It is an acute disease with symptoms ranging from mild, such as headache, sweating, hot flashes, piloerection, and anxiety, to severe [3–7], such as arrhythmia, including atrial fibrillation, and high systolic blood pressure (SBP) above 300 mmHg, which may lead not only to cerebral hemorrhage but also to convulsions and death [8–10].

Cardiovascular disease is one of the most common causes of death in patients with SCI. Moreover, the underlying causes of cardiovascular disease are due to abnormal blood pressure (BP) control such as AD and orthostatic hypotension (OH). A high prevalence of arrhythmia and abnormal cardiovascular responses after exercise are also important causes of cardiovascular disease [11]. In general, the diagnostic definition of AD is a rise in SBP by more than 20–40 mmHg [5]. However, considering that the baseline BP of patients with cervical or upper thoracic SCI has been reported to be lower than that of healthy persons by 15–20 mmHg [6, 7], normal or slightly elevated BP can be diagnosed as AD in patients with SCI. But one-time assessments, such as individual BP measurement, which are clinically practiced currently, or diagnosis depending on patients' symptoms, are not sufficient to make an accurate diagnosis of the various aspects of BP instability earlier in patients with SCI. Accordingly, a recent report proposing a method to assess such instability through long-term and real-time measurements of BP in patients with SCI using circadian BP profiles and ambulatory blood pressure monitoring (ABPM) has been shown to possess a good prognostic value for cardiovascular morbidity and mortality [12]. ABPM is an automated, noninvasive assessment method, which is useful for AD diagnosis and circadian BP assessment by continuous measurements of BP and heart rate (HR) during a period of 24 hours.

Therefore, in this study, we use the ABPM in patients with cervical or upper thoracic SCI above T6 in order to estimate the prevalence of AD by examining its incidence rate and patterns. Furthermore, the incidence rate of symptomatic or asymptomatic AD was examined using the autonomic dysfunction following spinal cord injury (ADFSCI) questionnaire [13] to propose the need for early diagnosis and interventions for asymptomatic AD.

## 2. Methods

### 2.1. Subjects

The subjects of this study were patients with SCI who were admitted to the rehabilitation department of Wonkwang University Hospital. The inclusion criteria for the subjects were as follows: (i) patients diagnosed with cervical or upper thoracic SCI above T6 via medical imaging or clinical assessment; (ii) patient age at least 19 years; (iii) patients without complications such as pneumonia and urinary tract infections; and (iv) neurologically stable patients who consented to participate in this study. Patients with serious or unstable physical diseases or patients with a past medical history of severe cardiovascular disease except hypertension were excluded.

The medical record of each patient including the demographic information (age, sex, cause of SCI, duration of SCI, etc.) was recorded and ABPM and ADFSCI questionnaire were administered. Neurological levels and SCI severity were determined using the American Spinal Injury Association Impairment Scale (AIS).

All studies were carried out in accordance with relevant standards after obtaining the approval of the Institutional Review Board (IRB number: 201601-HR-004) of Wonkwang University Hospital, and patients signed a written consent form prior to study participation after being informed about the purpose, methods, and other relevant information regarding the study.

### 2.2. Methods

#### 2.2.1. Functional Evaluation of the Autonomic Nervous System

For ABPM, a BR 102 plus (Schiller-Reomed AG, Baar, Zug, Switzerland) was attached to the nondominant upper limb of the patient using a cuff of an appropriate size, and SBP, diastolic BP (DBP), and HR were recorded within a 24-hour period. Collected data were analyzed using the MT-300 software. The 12-hour period between 7:00 a.m. and 7:00 p.m. was designated as daytime and the remaining time was designated as nighttime. SBP, DBP, and HR were measured every 30 minutes. Additionally, the patients were instructed to press the button for self-monitoring of BP whenever AD symptoms appeared.

AD was defined as SBP exceeding the baseline BP by 20 mmHg [2] and the baseline BP was obtained by calculating the averages of SBP measured during the 12 hours both in the daytime and in the nighttime. The frequency of AD was recorded as the number of incidences during the daytime and nighttime and also as the sum of daytime and nighttime incidences.

#### 2.2.2. Questionnaire of Autonomic Dysfunction following Spinal Cord Injury

To evaluate AD symptoms, AD criteria in Part III of the ADFSCI were used [13]. The evaluation criteria were the presence or absence and frequency of the following symptoms, which included headache, excessive sweating in the body above the area of SCI, goosebumps or anxiety, and palpitations. These were evaluated using a five-point scale system according to the degree of symptom severity (0: not at all; 5: very often, very severe) with a range of 0 ~ 204 points, and through this method, the frequency and severity of AD symptoms were recorded.

#### 2.2.3. Statistical Analysis

For statistical analysis, SPSS ver. 19.4 (IBM SPSS, Armonk, NY, USA) was used and the statistical significance was set at *p* < 0.05. Differences in the results of ABPM between daytime and nighttime were analyzed using the paired *t*-test and intergroup differences depending on the duration of symptoms and the presence or absence of AD symptoms were analyzed using the independent *t*-test.

## 3. Results

### 3.1. General Characteristics of the Subjects

The study included 22 men and 6 women with a mean age of 56.8 ± 10.5 (range: 31–77) years. Regarding the cause of SCI, 8 and 20 patients had SCI due to disease and trauma, respectively. SCI duration ranged from 7 days to 31 years and 7 months with an average duration of 28.4 ± 76.6 months. As for the distribution by duration of SCI, less than 3 months and longer than 3 months after injury were defined as the subacute stage and the chronic stage. There were 15 and 13 patients in the subacute and chronic groups, respectively. Regarding the level of injury, 24 patients had cervical SCI and 4 patients had thoracic SCI. With respect to SCI severity, 4 patients were classified as having motor complete SCI (AIS grades A and B) and 24 patients were classified as having motor incomplete SCI (AIS grades C and D) (Table 1).

### 3.2. Results of ABPM

The 24-hour SBP, DBP, and HR for all subjects were 120.6 ± 18.9 mmHg, 75.8 ± 10.7 mmHg, and 75.1 ± 13.4 bpm, respectively. The daytime SBP, DBP, and HR were 119.8 ± 18.8 mmHg, 75.6 ± 11.0 mmHg, and 76.3 ± 14.1 bpm, respectively, and the nighttime SBP, DBP, and HR were 123.8 ± 21.2 mmHg, 77.4 ± 11.1 mmHg, and 71.2 ± 13.1 bpm, respectively. It was observed that the nighttime SBP and DBP were higher than the daytime values, showing the loss of physiologic nocturnal dipping, and the nighttime HR was statistically significantly lower (*p* < 0.05). Twenty-six of the 28 patients (92.8%) had AD at least once and the average AD frequency was 2.7 ± 1.9 times during daytime and 3.1 ± 4.8 times during nighttime. The average AD frequency in 24 hours was 5.8 ± 4.7 times (Table 2). General characteristics of AD and non-AD groups were age (56.0 versus 61.0), male sex (75% versus 100%), cause of SCI (trauma = 66.7% versus 100%), and duration of SCI (32.6 versus 3.25). The percentage of cervical SCI was 83.3% versus 100% between AD and non-AD groups. The percentage of motor complete and motor incomplete SCI was 12.5% versus 25% between AD and non-AD groups.

### 3.3. Comparative Results by Duration of Disease

In the subacute group, SBP and DBP were 119.5 ± 19.4 mmHg and 74.4 ± 9.7 mmHg, respectively, in the daytime and 126.7 ± 22.8 mmHg and 77.2 ± 12.1 mmHg, respectively, in the nighttime. In the subacute group, nighttime SBP and DBP were higher than the corresponding daytime values, showing the loss of physiologic nocturnal dipping; however, in the chronic group, the absence of physiologic nocturnal dipping was not observed. In the subacute group, there was a statistically significant HR decrease in the nighttime at 66.4 ± 8.9 bpm compared with that in the daytime at 74.0 ± 12.3 bpm (*p* < 0.01); however, in the chronic group, there was no statistically significant difference in the HR between daytime and nighttime (*p* > 0.05).

The frequency of AD in the subacute group was 2.1 ± 1.8 times in the daytime and 3.6 ± 6.3 times in the nighttime, and the average frequency was 5.7 ± 5.8 times in 24 hours. In the chronic group, it was 3.4 ± 1.9 times in the daytime and 2.6 ± 2.2 times in the nighttime, and the average AD frequency was 6.0 ± 3.3 times in 24 hours (Table 3).

### 3.4. Comparative Results by the Severity of Injury

In the motor complete group, SBP and DBP were 102.5 ± 14.2 mmHg and 69.5 ± 13.2 mmHg, respectively, in the daytime and 103.50 ± 7.3 mmHg and 68.25 ± 6.1 mmHg, respectively, in the nighttime. In the motor incomplete group, SBP and DBP were 122.7 ± 18.1 mmHg and 76.6 ± 10.5 mmHg, respectively, in the daytime and 127.2 ± 20.8 mmHg and 78.9 ± 11.0 mmHg, respectively, in the nighttime. The nighttime SBP and DBP were higher than the corresponding daytime values, revealing the absence of physiologic nocturnal dipping in both groups. In the intergroup comparison, it was found that daytime, nighttime, and 24-hour SBP in the motor complete group were significantly lower than those in the motor incomplete group (*p* < 0.05).

There were no significant differences between daytime and nighttime HR in both groups (*p* > 0.05).

The frequency of AD was 3.5 ± 1.9 times in the daytime, 2.5 ± 3.0 times in the nighttime, and 6.0 ± 4.1 times in 24 hours in the motor complete group. In the motor incomplete group, it was 2.5 ± 1.8 times in the daytime, 3.25 ± 5.1 times in the nighttime, and 5.8 ± 4.9 times in 24 hours (Table 4).

### 3.5. Result of ADFSCI Questionnaire

The total scores of ADFSCI questionnaire ranged from 0 to 83 points with an average of 21.1 ± 29.6 points. Sixteen (57.1%) symptomatic patients had a total score of at least 1 point and twelve (42.9%) asymptomatic patients had a total score of 0 points. General characteristics of AD and non-AD groups were age (53.3 versus 59.7), male sex (69% versus 86.7%), cause of SCI (trauma = 84.6% versus 60.0%), and duration of SCI (20.2 versus 35.5). The percentage of cervical SCI was 92.3% versus 80.0% between AD and non-AD groups. The percentage of motor complete SCI was 23.1% versus 6.7% between AD and non-AD groups.

### 3.6. Comparison of ABPM Results Depending on the Presence or Absence of Symptoms

In the symptomatic group, SBP and DBP were 124.7 ± 25.2 mmHg and 76.6 ± 13.3 mmHg, respectively, in the daytime and 131.2 ± 26.6 mmHg and 80.3 ± 14.6 mmHg, respectively, in the nighttime. SBP and DBP were higher at nighttime with a loss of the physiologic nocturnal dipping. In the asymptomatic group, SBP and DBP were 123.1 ± 13.8 mmHg and 77.9 ± 12.2 mmHg, respectively, in the daytime and 122.1 ± 19.9 mmHg and 75.9 ± 10.1 mmHg, respectively, in the nighttime. The loss of the physiologic nocturnal dipping was not observed in the asymptomatic group. In the intergroup comparison, daytime, nighttime, and 24-hour SBP tended to be higher in the symptomatic group than in the asymptomatic group, although no statistical significance was observed (*p* > 0.05).

No significant difference in HR was observed between daytime and nighttime in both groups (*p* > 0.05). The frequency of AD was 2.7 ± 2.0 times in the daytime, 3.0 ± 2.9 times in the nighttime, and 5.7 ± 3.5 times in 24 hours in the symptomatic group and was 3.3 ± 1.7 times in the daytime, 2.7 ± 3.0 times in the nighttime, and 6.0 ± 3.3 times in 24 hours in the asymptomatic group (Table 5).

## 4. Discussion

Autonomic nervous system disorders after SCI are common, among which AD and OH mainly occur due to the malfunction of the cardiovascular system. Cardiovascular complications have been reported to be the most common cause of death in patients with SCI, and the proposed causes include not only the metabolic syndrome, which consists of obesity, lipid metabolic disorders, and diabetes, but also a reduction of daily energy consumption due to reduced physical activity [14–16]. In addition, a high prevalence of cardiovascular disorders, such as abnormal BP control and arrhythmias, and abnormal cardiovascular responses after exercise have also been suggested as important causes [11]. Despite the high prevalence of abnormal BP control in patients with SCI, currently, evaluations are only restricted to symptoms and signs that appear in each individual patient with SCI at the time of hypotensive and hypertensive crises. Furthermore, there is no accurate report of AD and OH prevalence in South Korea, as determined using accurate assessment tools.

While the prevalence of AD in patients with SCI has been variously reported to range from 20 to 70%, it is known to occur in more than 90% of patients with cervical or upper thoracic SCI [17, 18]. In general, the diagnostic definition of AD is a rise in SBP by more than 20–40 mmHg [2]. However, considering that the stabilized BP of patients with cervical or upper thoracic SCI is reported to be lower than that of healthy persons by 15–20 mmHg [8, 19], normal or slightly elevated BP can be diagnosed as AD. Thus, in this study, we intended to determine the prevalence rate of AD using ABPM and the ADFSCI questionnaire in patients with cervical and upper thoracic SCI. According to a previous study [13], ABPM is a useful test for assessing BP instability and fluctuation of BP in the daytime and nighttime in SCI patients with AD. According to the results of this study, the prevalence rate of AD in patients with cervical and upper thoracic SCI, which was determined using ABPM, was 92.8%, and this result was consistent with the previous study. According to the results of the ADFSCI questionnaire in this study, AD prevalence was 57.1%, which was similar to a previous study which showed that AD symptoms occurred in about 70% of patients [20].

This typical daily BP pattern in SCI patients is characterized by the occurrence of potentially life-threatening hypertensive events due to AD as well as hypotensive events [21]. These immense BP fluctuations could contribute to increased shear stress of blood vessels and eventually to vascular injury, predisposing this population to a greater risk of arterial disease [22]. Although the impact of AD on the prevalence of cardiovascular and cerebrovascular diseases is not fully clarified, considering the report that cardiovascular instability in patients with SCI increased the frequency of heart disease and ischemic stroke occurrence [23], appropriate BP control and evaluation of cardiovascular instability are important. In addition, inappropriate activation of the sympathetic nervous system, which is associated with AD, occurs several times a day and could be asymptomatic in spite of the significantly elevated arterial BP [9, 24]. Thus, it is necessary to diagnose such asymptomatic AD earlier on and apply appropriate treatment interventions. In this present study, although the risk of morbidity from heart disease or cerebral vascular disease in 35.7% of the patients who were diagnosed with asymptomatic AD is likely to increase significantly, it was difficult to diagnose such asymptomatic AD using one-time BP measurements. Therefore, it is suggested that, as a preventive measure for cardiovascular disease in patients with cervical and upper thoracic SCI, early diagnosis and therapeutic interventions for AD are required using ABPM in the early stage. Medical personnel, caregivers, and individuals with SCI should be aware of the importance of the timely diagnosis and management of this life-threatening condition, which can result in a variety of significant complications including stroke, seizures, myocardial ischemia, and death [25].

It has been reported that the prevalence of AD increases according to the level and severity of SCI [20, 26, 27]. A previous study reported that AD occurred in 91% of patients with complete quadriplegia and only in 27% of patients with incomplete injury [20]. Based on the results of ABPM, in this study, AD was observed in 100% and 91.6% of patients with motor complete and motor incomplete SCI, respectively. This differs from the previous report on the prevalence of AD in incomplete injury, because the previous report was about the prevalence of AD during the urodynamic study in chronic SCI patients. Moreover, in the intergroup comparison, significant differences in daytime, nighttime, and 24-hour SBP were observed, in which AD occurred more frequently in the motor complete group with a concomitant increase in the number of AD events.

The timing of AD onset is not yet clear. Although previous researchers believed that AD was only limited to chronic SCI, recent reports show AD occurrence one day or several weeks after SCI [27, 28]. These results suggest that patients with acute and subacute SCI were not diagnosed early and did not receive appropriate treatment for AD. Moreover, the relationships between the level and severity of injury and the timing of AD onset are currently not clear.

The phenomenon where SBP and DBP decrease at nighttime by at least 10% compared with daytime values is defined as physiological nocturnal BP dipping [29] and is reportedly the most sensitive predictor of cardiovascular disorders [12]. In patients with SCI, the absence of this nocturnal BP dipping or even the elevation of nocturnal BP has been observed. According to the results of the ABPM analysis performed in this study, in which the patients were divided into subacute and chronic stages by setting 3 months as the cut-off of the disease onset, it was found that the nighttime SBP in the subacute group was higher by 7 mmHg compared to the chronic group, and this result was consistent with a previous report of nocturnal BP dipping loss in patients with SCI [12]. The subacute group showed a significant difference in the daytime and nighttime SBP (*p* < 0.05) with a concomitant loss of nocturnal BP dipping and a significant decrease in the nighttime HR (*p* < 0.01). However, the chronic group showed no significant differences in nocturnal BP dipping and HR. Considering the report that significant morphological changes such as atrophy of sympathetic preganglionic neurons and loss of dendritic arbors were observed in the acute phase [30], it is believed that neural regeneration-mediated recovery of the autonomic nervous system by passage of time or acclimatization of patients to the stimuli and symptoms, which cause AD and acquisition of self-control ability [9], influenced the above results.

Limitations of this study were (1) the small sample size of the subjects, (2) the incomplete exclusion of causes that may induce AD, and (3) the fact that although the relationship between activities and AD onset was analyzed by recording activities of daily living in the daytime, the application of this type of evaluation, which is based on the record of patients or caregivers, is limited due to its lack of accuracy. Therefore, additional research addressing these limitations is necessary.

## 5. Conclusion

The results of this study showed that the incidence rate of AD was 92.8% in patients with cervical and upper thoracic SCI and the incidence rate of asymptomatic AD was 42.9%. To manage long-term cardiovascular diseases and to lower mortality in patients with SCI, aggressive diagnosis and therapeutic interventions for AD using ABPM in the early stage should be considered even in asymptomatic patients.

## Figures and Tables

**Table 1:** General characteristics of patients with spinal cord injury (*n* = 28).

Demographic factor	Value
Gender (male : female)	22 : 6
Mean age (yr)	56.8 ± 10.5
Etiology (spontaneous : traumatic)	8 : 20
Lesion duration (mo)	28.4 ± 76.6
Subacute	15
Chronic	13
Level of spinal cord injury	
Cervical	24
Thoracic	4
ASIA scale	
Motor complete (grades A and B)	4
Motor incomplete (grades C and D)	24

Values are presented as mean ± standard deviation or number. ASIA: American Spinal Injury Association.

**Table 2:** Results of ABPM in patients with spinal cord injury.

	Daytime	Nighttime	24 h
SBP	119.9 ± 18.8	123.8 ± 21.2	120.6 ± 18.9
DBP	75.6 ± 11.0	77.4 ± 11.1	75.8 ± 10.7
HR	76.3 ± 14.1	71.2 ± 13.1^*∗*^	75.1 ± 13.4
AD event	2.7 ± 1.9	3.1 ± 4.8	5.8 ± 4.7

Values are presented as mean ± standard deviation or number. ^*∗*^*p* < 0.05, significant difference between the daytime values and the nighttime values. SBP: systolic blood pressure; DBP: diastolic blood pressure; HR: heart rate; AD: autonomic dysreflexia.

**Table 3:** Comparison of ABPM values in subacute and chronic SCI.

	Daytime	Nighttime	24 h
SBP			
* Subacute*	119.5 ± 19.4	126.7 ± 22.8^*∗*^	120.6 ± 19.7
* Chronic*	120.3 ± 18.9	120.5 ± 19.5	120.4 ± 18.7
DBP			
* Subacute*	74.4 ± 9.7	77.2 ± 12.1	74.4 ± 10.1
* Chronic*	77.0 ± 12.6	77.6 ± 10.2	77.3 ± 11.4
HR			
* Subacute*	74.0 ± 12.3	66.4 ± 8.9^*∗∗*^	72.4 ± 11.1
* Chronic*	78.8 ± 16.1	76.8 ± 15.2^†^	78.1 ± 15.5
AD event			
* Subacute*	2.1 ± 1.8	3.6 ± 6.3	5.7 ± 5.8
* Chronic*	3.4 ± 1.9	2.6 ± 2.2	6.0 ± 3.3

Values are presented as mean ± standard deviation or number. ^*∗*^*p* < 0.05 and ^*∗∗*^*p* < 0.01, significant difference between the daytime values and the nighttime values. ^†^*p* < 0.05, significant difference between subacute and chronic SCI. SBP: systolic blood pressure; DBP: diastolic blood pressure; HR: heart rate; AD: autonomic dysreflexia.

**Table 4:** Comparison of ABPM values between motor complete and incomplete SCI.

	Daytime	Nighttime	24 h
SBP			
* Complete*	102.5 ± 14.2	103.5 ± 7.3	102.5 ± 11.0
* Incomplete*	122.7 ± 18.1^†^	127.2 ± 20.8^†^	123.5 ± 18.3^†^
DBP			
* Complete*	69.5 ± 13.2	68.2 ± 6.1	69.2 ± 10.7
* Incomplete*	76.6 ± 10.5	78.9 ± 11.0	76.8 ± 10.5
HR			
* Complete*	73.2 ± 10.2	72.7 ± 9.5	73.5 ± 10.3
* Incomplete*	76.7 ± 14.7	71.0 ± 13.7	75.3 ± 14.0
AD event			
* Complete*	3.5 ± 1.9	2.5 ± 3.0	6.0 ± 4.1
* Incomplete*	2.5 ± 1.8	3.25 ± 5.1	5.8 ± 4.9

Values are presented as mean ± standard deviation or number. ^*∗*^*p* < 0.01, significant difference between the daytime values and the nighttime values. ^†^*p* < 0.05, significant difference between motor complete and incomplete SCI. SBP: systolic blood pressure; DBP: diastolic blood pressure; HR: heart rate; AD: autonomic dysreflexia.

**Table 5:** Comparison of ABPM values between symptomatic and asymptomatic AD.

	Daytime	Nighttime	24 h
SBP			
* Symptomatic*	124.7 ± 25.2	131.2 ± 26.6	126.7 ± 25.1
* Asymptomatic*	123.1 ± 13.8	122.1 ± 19.9	122.5 ± 14.5
DBP			
* Symptomatic*	76.6 ± 13.3	80.3 ± 14.6	77.7 ± 13.2
* Asymptomatic*	77.9 ± 12.2	75.9 ± 10.1	77.1 ± 11.3
HR			
* Symptomatic*	78.8 ± 14.7	74.2 ± 14.4	77.6 ± 14.4
* Asymptomatic*	75.9 ± 15.2	69.7 ± 14.8	74.5 ± 14.4
AD event			
* Symptomatic*	2.7 ± 2.0	3.0 ± 2.9	5.7 ± 3.5
* Asymptomatic*	3.3 ± 1.7	2.7 ± 3.0	6.0 ± 3.3

Values are presented as mean ± standard deviation or number. SBP: systolic blood pressure; DBP: diastolic blood pressure; HR: heart rate; AD: autonomic dysreflexia.
